# Towards renewables development: Review of optimization techniques for energy storage and hybrid renewable energy systems

**DOI:** 10.1016/j.heliyon.2024.e37482

**Published:** 2024-09-10

**Authors:** Oluwatoyosi Bamisile, Dongsheng Cai, Humphrey Adun, Mustafa Dagbasi, Chiagoziem C. Ukwuoma, Qi Huang, Nathan Johnson, Olusola Bamisile

**Affiliations:** a College of Nuclear Technology and Automation Engineering, Chengdu University of Technology, Sichuan Engineering Technology Research Centre for Industrial Internet Intelligent Monitoring and Application, Sichuan, 610059, PR China; b Energy Systems Engineering Department, Cyprus International University, Haspolat-Lefkosa, Mersin 10, Turkey; c Centre for Environmental Policy, Imperial College London, United Kingdom; d Energy and Environmental Science Division, CEPMLP, University of Dundee, Scotland, United Kingdom

**Keywords:** *Optimization*, *Hybrid energy System*, *Energy storage*, *Energy storage techniques*, *Intermittent energy sources*

## Abstract

As global energy demand and warming increase, there is a need to transition to sustainable and renewable energy sources. Integrating different systems to create a hybrid renewable system enhances the overall adoption and deployment of renewable energy resources. Given the intermittent nature of solar and wind, energy storage systems are combined with these renewable energy sources, to optimize the quantity of clean energy used. Thus, various optimization strategies have been developed for the integration and operation of these hybrid renewable energy systems. Existing studies have either reviewed hybrid renewable energy systems or energy storage systems, however, these studies ignored energy storage systems integrated with hybrid renewable energy systems. This study offers a comprehensive analysis of the optimization methods used in hybrid renewable energy systems (HRES) integrated with energy storage systems (ESS). We examined the optimization models used in the integration of HRES and ESS, their objectives, and the common constraints. Based on our review, capacity and CO_2_ emissions constraints were frequently used in hybrid optimization techniques that are effective approaches for integrating HRES and ESS. This research supports the move towards sustainable, clean energy solutions by combining an analysis of energy storage techniques with the optimization of hybrid renewable energy systems.

**Table:** NOMENCLATURE

***Abbreviations***	***Meaning***
AC	Alternate Current
A-CAES	Adiabatic Compressed Air Energy Storage
ANN	Artificial Neural Network
ATES	Aquifer Thermal Energy Storage
BES	Battery Energy Storage
BTES	Borehole Thermal Energy Storage
CAES	Compressed Air Energy Storage
CES	Chemical Energy Storage
CUF	Capacity Utilization Factor
DC	Direct Current
DOD	Depth of Discharge
ECES	Electrochemical Energy Storage
EENS	Expected Energy Not Supplied
EES	Electrical Energy Storage
EGR	Energy Generation Ratio
EIR	Energy Index of Reliability
ELF	Equivalent Loss Factor
EMR	Electricity Match Rate
ESS	Energy Storage Systems
EUE	Expected Unserved Energy
ExCF	Exegetic Capacity Factor
FEE	Final Excess Energy
FES	Flywheel Energy Storage
FBES	Flow Battery Energy Storage
FLNS	Fractional Load Not Served
GA	Genetic Algorithms
GES	Gravity Energy Storage
HRES	Hybrid Renewable Energy System
HWTES	Hot Water Thermal Energy Storage
LA	Level of Autonomy
LHS	Latent Heat Storage
LOEE	Loss of Energy Expectation
LOHE	Loss of Healthy Expectation
LOLE	Loss of Load Expectation
LOLH	Loss of Load Hours
LOLP	Loss of Load Probability
LOLR	Loss of Load Risk
LOPSP	Loss of Power Supply Reliability
MCS	Monte Carlo Simulations
MEES	Maximum Expected Energy Supplied
MSTES	Molten Salt Thermal Energy Storage
PCM	Phase Change Materials
PDF	Probability Density Function
PHES	Pumped Hydro Energy Storage
PSO	Particle Swarm Optimization
PV	Photovoltaic
PTES	Pumped Thermal Energy Storage
REF	Renewable Energy Fraction
RPS	Reliability of Power Supply
SAIDI	System Average Interruption Duration Index
SHS	Sensible Heat Storage
SMES	Superconducting Magnetic Energy Storage
SNG	Synthetic Natural Gas
SPL	System Performance Level
TCES	Thermochemical Energy Storage
LGBM	Light Gradient Boosting Machine
ADABOOST	Adaptive Boosting
*Symbols*	
CO_2_	Carbon Dioxide

## 1 Introduction

The increase in global energy consumption is a consequence of rapid industrialization, technological advancements, and robust economic growth globally [1]. Rising energy demand has led to an increased focus on renewable energy sources. Renewable energy systems, such as solar, wind, geothermal, and tidal power, offer a sustainable alternative, providing clean energy solutions that reduce greenhouse gas emissions and dependence on fossil fuels [216].

**Fig. 1:**
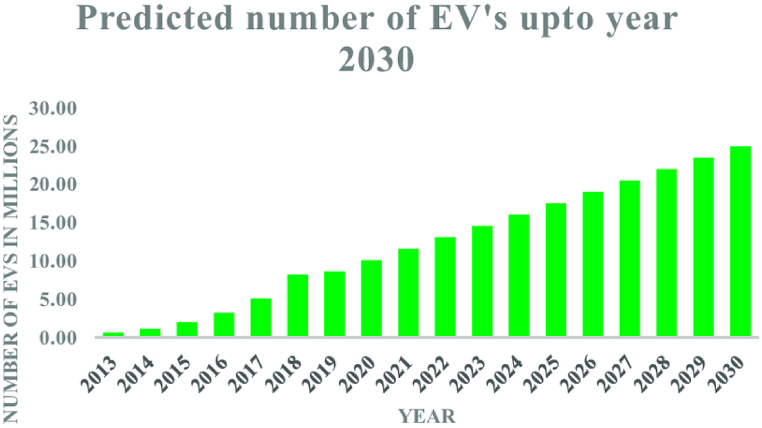
Past and projected future deoloyment 1of Electric Vehicles in the global market [6].

The adoption of clean technologies is evident as the number of electric cars on the road has increased nearly tenfold in the last 10 years as seen in Fig. 1. Renewable energy sources accounted for 30% of the world's electricity mix in 2023 [2]. Globally, electric heating systems such as heat pumps are outselling fossil fuel boilers, and new offshore wind projects are attracting three times the investment of new coal- and gas-fired power plants [3]. However, it's crucial to acknowledge that renewable resources are intermittent [4], and do not follow demand profiles which vary diurnally, seasonally and regionally [218]. For instance, wind turbines' capacity factor can drop to zero in calm conditions while solar panels' capacity factor declines to zero during dark hours [5].

**Fig. 2:**
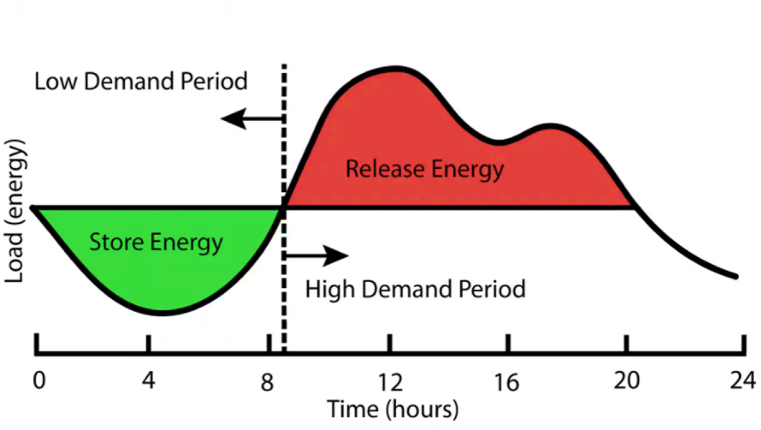
An exemplary 24-hour charge-discharge cycle of an energy storage system [9].

To maintain a balance between intermittent renewable energy resource production and consumption, energy storage systems (ESS) are required [7]. ESS holds significant potential for optimizing energy management and cutting down on energy waste caused by curtailment. These systems vary in their design, each aimed at gathering energy from diverse sources and storing it for a range of applications [8]. Fig. 2 shows the charge-discharge cycle of an ESS over 24 hours. Energy is stored during low demand as represented by the green area under the curve, while during high demand periods, energy is released as represented by the red area above the curve.

Hybrid Renewable Energy Systems (HRES) are energy systems that combine multiple renewable energy sources to enhance reliability and efficiency [10]. By integrating diverse sources, HRES can mitigate the limitations of a single renewable technology, ensuring a more consistent energy supply [[11], [12], [13]]. HRES finds application in diverse settings, including universities, hospitals, airports, corporate offices, and commercial and business districts. Notably, data centres, known for their substantial energy consumption greatly benefit from HRES. For data centres, HRES provides a reliable and environmentally friendly energy supply for power and cooling requirements. Moreover, HRES effectively utilize the significant waste heat produced by data centres, enhancing overall system efficiency and cost-effectiveness [14].

To maximize the effectiveness and efficiency of HRES across various applications, it is important to optimize system operations and costs [15]. This involves selection of energy sources, types, and capacities, as well as the formulation of operational strategies [16]. Given the multifaceted nature of HRES, characterised by various components, diverse structural configurations, and flexible operational strategies, configuring and optimizing these systems poses challenges for maintenance and reliability, structural configuration, techno-economics, and operational strategy [17]. Consequently, extensive research has been conducted in this field.

Optimization comprises three primary components: efficiency assessment, model development, and model solution. In recent years, several evaluation criteria, such as economic, efficiency, and environmental metrics, have served as the basis for key energy decisions [18,19]. The development of the model closely intertwines with the nature of the study and research requirements. In addition to devising an objective function largely based on efficiency outcomes, the representation of other constraints is important [20,21]. These constraints encompass a range of non-physical limitations, including financial constraints and human preferences [22,23], in addition to internal system constraints and physical interactions between the system and its surrounding environment [24]. Equally vital is the attention devoted to the model's solution. Hybrid energy systems typically feature complex optimization models, which pose challenges in developing efficient solution algorithms. Numerous approaches to problem resolution have been proposed, including classical algorithms [25], intelligent algorithms [26], and hybrid algorithms [27].

This paper reviews the optimization techniques for HRES, with a focus on studies that integrate ESS with HRES. While previous review papers focused on energy storage, hybrid renewable energy sources, or optimization approaches for each domain, to the best of our knowledge, none have thoroughly explored the optimization of energy storage technologies integrated with HRES. We categorize the optimization techniques into three groups, namely conventional, new generation, and hybrid techniques to map out the differences and similarities across studies. This study is important because the integration of ESS with HRES significantly enhances their reliability and efficiency. 10.13039/100014337Furthermore, optimizing these integrated systems contributes to sustainable energy solutions, and supports global efforts towards achieving energy security and reducing carbon emissions. This study emphasizes using optimization methodologies to assess cost minimization, system efficiency and reliability and highlights common optimization techniques and renewable energy combinations. It serves as a foundation for future research, offering insight into typical objective functions and constraints in specific models.

The paper is divided into six sections. Section 2 explains the methodological approach used. Section 3 provides an overview of the integrated HRES structure, and Section 4 reviews energy storage technologies. The optimization methods employed in HRES and ESS are reviewed in Section 5, and Section 6 explores the key criteria and constraints influencing the optimization strategies. Finally, Section 7 summarizes the key findings and insights gained from the literature review.

## 2 Materials and methods

**Fig. 3:**
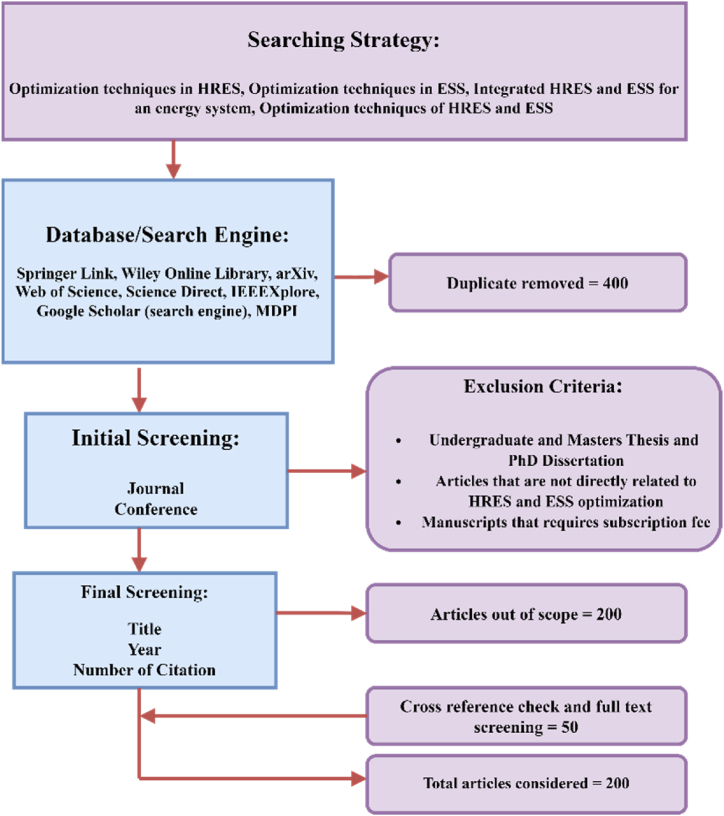
Schemativ representation of the PRISMA model strategy.

This study presents a comprehensive review of the optimization techniques employed in HRES integrated with energy storage systems. The methodological approach used in selecting the academic materials reviewed in this paper is the Preferred Reporting Items for Systematic Reviews and Meta-Analyses (PRISMA) model strategy and is summarized in Fig. 3. The PRISMA model strategy is a tool used for documenting each stage of the literature search process across multiple resources, clearly showing the progression from initial database searches to the final selection of papers for review [28].

A literature search was conducted across multiple databases and search engines, including Springer Link, Wiley Online Library, Science Direct, IEEEXplore, and Google Scholar. The initial screening focused on identifying relevant journal articles and conference papers related to HRES and ESS optimization. To ensure the rigour and relevance of the review, the study applied a set of exclusion criteria. Undergraduate and master's theses, articles not directly focused on HRES and 10.13039/501100004895ESS optimization, and manuscripts that required subscription fees for access were excluded.

After the initial screening, the final review process considered factors such as the title, publication year, and number of citations of the remaining articles. This allowed the researchers to identify the 200 most relevant and impactful studies on the topic. The final set of articles was then subjected to a cross-reference check and full-text screening to ensure the completeness and accuracy of the review.

## 3 Renewable energy systems

Renewable energy sources use energy flows present in our natural environment to generate electricity, based on resources that are sustainable over the long term [29]. These sources include geothermal energy, wind power, oceanic wave and tidal energy, hydropower, biofuels, and solar energy obtained directly from the sun, as depicted in Table 1. .

**Table 1:** Summary of different renewable energy systems.

References	Renewable Energy System	Summary
[[30], [31], [32]]	Solar Energy	Recent developments in solar energy systems involve cutting-edge technologies such as bifacial solar panels, which efficiently harness sunlight from both sides, resulting in enhanced energy production. Floating solar farms utilize photovoltaic panels placed on water surfaces, thereby maximizing land efficiency and minimizing water evaporation. Solar windows incorporate solar cells into transparent materials, thereby converting windows into sources of energy. Lenses are used by Concentrated Solar Power (CSP) to focus sunlight upon a small area, creating intense heat to generate energy.
[[33], [34], [35]]	Wind Energy	Wind energy systems utilize the kinetic energy of air in motion to produce electrical power. Conventional wind turbines transform the movement of wind into rotational energy, which in turn powers generators. Recent developments involve the establishment of offshore wind farms that take advantage of the higher and more reliable winds found over the ocean. Vertical-axis wind turbines provide flexibility in their design and are utilized in a wide range of environments. Intelligent wind turbines employ sensors and data analytics to achieve optimal performance.
[[36], [37], [38]]	Bioenergy	Bioenergy systems employ organic substances such as biomass, crops, or waste to generate energy. Conventional approaches encompass the use of wood as a fuel source, whilst contemporary techniques incorporate the conversion of biomass into biofuels or the production of power through combustion or anaerobic digestion. Recent developments include the production of sophisticated biofuels derived from algae or cellulosic feedstocks, which improve fuel economy and mitigate emissions. Biogas systems harness methane emissions from biological waste, offering a sustainable and environmentally friendly energy alternative. Ongoing research is centred on sustainable raw materials, conversion methods, and integrated bioenergy solutions to improve both the environmental and economic advantages.
[39,40]	Ocean Energy	Ocean energy systems conventionally extract energy from tides and waves Tidal stream generators employ subaquatic turbines to transform tidal currents into electrical energy. Wave energy converters harness the energy derived from oceanic waves. Ocean thermal energy conversion (OTEC) utilizes the disparity in temperature between the uppermost layer of the ocean and its deeper regions.
[41,42]	Hydropower	Hydropower systems utilize the kinetic energy of moving or descending water to produce electrical power. Conventional dams and turbines have been utilized for a significant period. Run-of-river hydropower systems mitigate environmental effects by facilitating the natural flow of water. Pumped storage hydropower ensures system stability by accumulating surplus energy during periods of low demand.
[43]	Geothermal Energy	Geothermal energy systems utilize thermal energy derived from the Earth's subsurface to provide energy. Conventional techniques entail the extraction of steam or hot water from reservoirs to generate energy. Recent developments involve the use of enhanced geothermal systems (EGS), which employ hydraulic fracturing to establish permeability in high-temperature rocks. Binary cycle power plants effectively produce electricity from geothermal resources with lower temperatures. The adaptability of geothermal energy is demonstrated by its direct-use applications, such as heating houses.
[44,45]	Hybrid Renewable Energy	Hybrid renewable energy systems combine various sustainable energy sources to improve effectiveness and dependability. By integrating solar and wind power, it is possible to increase the reliability and consistency of energy production. Battery storage systems accumulate surplus energy for subsequent utilization, improve the stability of the power grid. Intelligent microgrid solutions enhance the efficiency of managing different energy sources. Recent technological progress has led to the development of hybrid systems that combine solar, wind, and energy storage, resulting in robust and flexible solutions.

While wind energy uses turbines to turn kinetic energy into electricity [46], solar energy is absorbed through photovoltaic systems [46] and concentrating solar power [47]. Bioenergy, which comes from biological materials and has a variety of sources, including agricultural and forest leftovers, is used for transportation, heating, and electricity generation. Ocean energy uses the abundant energy from surface waves, ocean currents, and temperature changes. It includes tidal and wave energy [48]. The movement of water from higher to lower altitudes produces hydropower, which is a major source of electricity globally, one clean, ecologically friendly way to generate power is through hydropower [49,50]. However, social and environmental repercussions, such as relocation and altered river ecosystems, need to be considered [51]. Geothermal energy provides a sustainable source of heat and electricity by drawing energy from the Earth's internal heat sources, which come from either upgraded geothermal systems or naturally occurring hydrothermal reservoirs [52].

Theoretically, worldwide biomass technology has the potential to annually produce 3500 EJ. The annual technical potential of hydropower stands at 14,576 TWh, yet we must consider social effects such as displacement and alterations to river habitats [53]. Hydrothermal reservoirs and enhanced geothermal systems can harness geothermal energy by tapping into the Earth's internal heat sources. Renewable energy systems are essential in the global search for cleaner alternatives. To optimize their benefits and minimize their negative effects on the environment and society, they require constant study and careful management [52].

### 3.1 Hybrid renewable energy systems

**Fig. 4:**
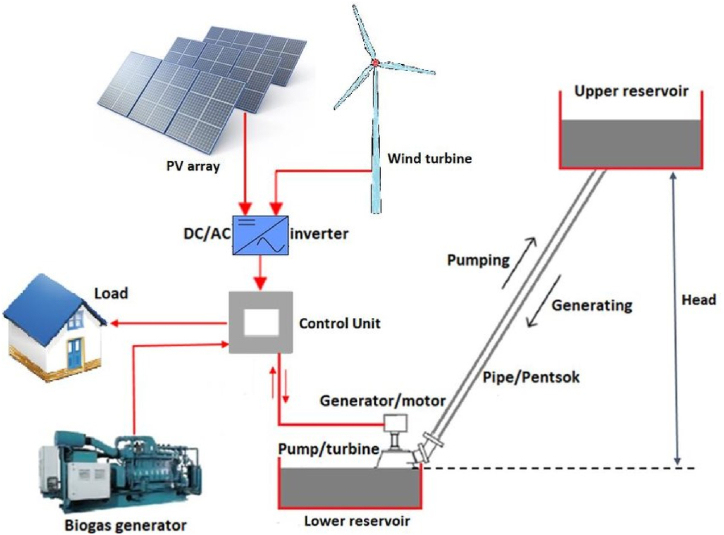
An exemplary Hybrid Renewable Energy System [55].

Weather conditions like solar irradiance and wind speed influence renewable energy sources. This inherent variability makes them unreliable and intermittent, unable to offer a consistent power supply. A solution to this is integrating various renewable energy sources, including fuel cells, solar, wind, biogas, and hydropower into a hybrid system. For instance, wind turbines are often integrated with hydropower, biomass energy or solar panels to provide a more constant and dependable power supply [54]. Fig. 4 introduces the concept of a hybrid energy system, which integrates multiple energy sources, whether renewable or non-renewable.

The system's core components include a photovoltaic (PV) array and a wind turbine. The DC/AC inverter connects the PV array and wind turbine, converting the DC power they produce into AC power—a prevalent form of electrical energy. This inverter interfaces with a control unit, a pivotal element of the hybrid energy system, which manages the distribution of power to different components by receiving the AC power from the DC/AC inverter. The control unit is connected to a load, which represents the devices or equipment that consume electrical energy. These loads include appliances, machinery, or any other electrical devices. The biogas generator is another energy source in the system. It generates electricity from biogas, which is produced through the decomposition of organic materials. The biogas generator is connected to the control unit. The generator/motor is connected to the control unit, used for backup power generation or energy storage purposes. There is a pump/turbine connected to a lower reservoir. The pump/turbine has a two-way is used for pumping water and generating electricity. During periods of excess electricity, operators pump water to the upper reservoir. Conversely, during high-demand periods for electricity, they release the stored water to generate power via the turbine [56].

### 3.2 Integrated structure of HRES

Integrated structures are crucial for optimizing the performance and efficiency of HRES. Zhang et al. [57] classified the integration methods for HRES into two primary categories: series integration and parallel integration [57]. The two energy sources in the case of series integration do not function independently; instead, they are intricately coupled inside the same system and function jointly while in the case of parallel integration, the two energy sources operate independently within the system.

#### 3.2.1 Series integration

**Fig. 5:**
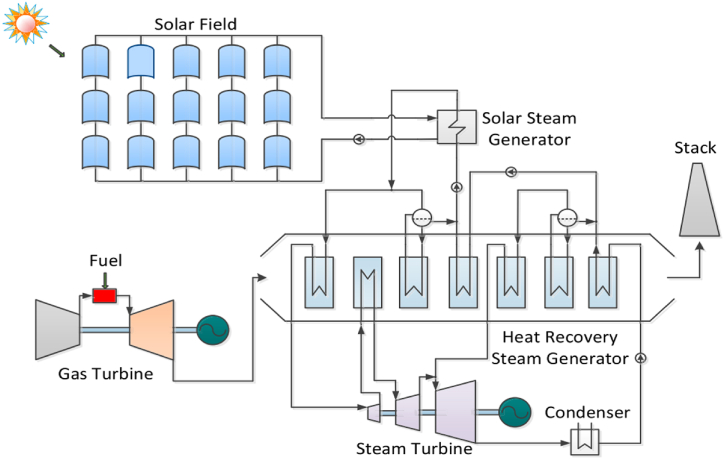
Exemplary series-integrated solar-gas multi-energy system [57].

In the context of hybrid energy systems, series integration involves systematically incorporating different energy sources or components throughout the conversion process. This integration arranges components in a specific sequence, typically one following another. The combination of solar energy and natural gas appears as a common strategy in the context of hybrid energy systems and is shown in Fig. 5.

Solar energy plays a role in the synthesis and decarbonization of gaseous fuels in addition to the integrated setup [58]. In this configuration, natural gas mixes with water vapour at high temperatures produced by solar energy, producing H_2_ and CO_2_. A H_2_-rich fuel is then produced by eliminating CO_2_ from the mixture. Zhang et al. [57], show that this procedure not only boosts gas turbine-powered efficiency to 39.2%, marking a notable 7.9% gain over reference systems, but also reduces 92% of CO_2_. Another study [59] harnesses solar energy to generate syngas. Subsequently, when integrated with natural gas, the synthesized gas fuels a gas turbine and generates energy. Another study demonstrates that utilizing all syngas as fuel reduces natural gas consumption by 20% [58].

Integration of solar energy with gas turbines is discussed in another study [60]. Solar energy was used to heat air leaving a compressor, mixed with hot exhaust gases, and fed to a turbine for electricity generation. This approach surpasses traditional coupling with natural gas and qualifies as a pure solar system [61]. In their setup, a solar collector heats a portion of the air leaving the regenerator and then channels it into the combustion chamber, where the solar collector acts as a secondary heat source for the air. Besides standalone power generation systems, solar energy frequently integrates into combined cooling, heating, and power systems or combined heat and power (CHP) plants. Yagoub et al. [62], examined the performance of a system that integrates solar energy with a gas boiler and a turbine cogeneration unit. They used solar energy to warm the boiler flue gas, which is then merged with the high-temperature exhaust gases from the combustion chamber to heat the working fluid.

#### 3.2.2 Parallel integration

**Fig. 6:**
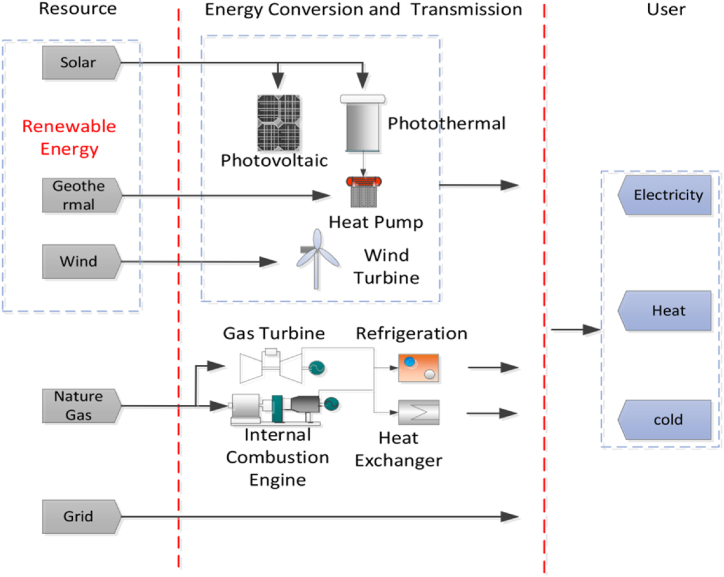
Exemplary parallel-integrated multi-energy system [57].

Parallel integration, in the context of a hybrid energy system, refers to the simultaneous and coordinated operation of multiple energy sources on a common platform as seen in Fig. 6. This approach allows different energy generation components, such as wind turbines, solar panels, microturbines, and energy storage systems, to work together to meet the overall energy demands of a system. Unlike series integration, where different energy sources are integrated sequentially, parallel integration enables these sources to operate concurrently, offering greater flexibility, efficiency, and the ability to optimize capacity and control for various user needs, including heating, cooling, and power. One study [63] demonstrates that green energy sources significantly reduce carbon emissions associated with natural gas power networks. This study utilized the CAM tool to construct a Distributed Energy Resources (DER) model integrating solar panels, natural gas-based cooling, heating, and power generation. The primary objectives of the optimization target were to minimize CO_2_ emissions and achieve the lowest cost. Incorporating solar energy led to a notable 72% reduction in CO_2_ emissions, despite an overall increase in the cost of electricity.

Additionally, the classification of hybrid energy systems considers the forms of energy sources within the system. For instance, hybrid systems encompass carbon and non-carbon sources, as exemplified by carbon hybrid power systems [64,65], PV-wind hybrid systems [66], PV-wind-diesel-battery hybrids [67], and hybrid systems combining natural gas and compressed air storage [68]. The categorization is further refined based on the relative significance or contribution of each power source within the system, as observed in coal-based power generation [64], the integration of solar thermal energy with gas turbines [69], and hybrid power systems where natural gas serves as the primary fuel [70].

### 3.3 Control strategies for hybrid renewable energy systems

Efficient control strategies play a pivotal role in maximizing the performance of hybrid renewable energy systems. Numerous research studies have explored specific setups and methodologies to enhance the reliability and efficiency of these systems. One experimental study underscored the importance of aligning the diesel engine's rated power with peak demand. This study investigated a hybrid PV and diesel engine system operating under constant loads without an energy storage system. Simulations demonstrated the system's effectiveness, particularly when radiation and load conditions varied, underscoring the necessity for control measures [71]. Another research project focused on modeling and managing a complete hybrid power system comprising photovoltaic, wind, and fuel cells [72]. Their objective was to provide continuous power for an electric vehicle through critical equipment design, parameter identification, and thorough subsystem analysis. The study's contribution lies in the development of mathematical models and power management techniques, including the incorporation of a battery bank [73]. Furthermore, a distinct hybrid system incorporating fuel cells, photovoltaics, and wind power was modelled in another study in the literature [74]. It featured a static variable regulator for output voltage control. The system's capability to endure harsh operating conditions was demonstrated by the intelligent controller, equipped with algorithms for optimizing turbine speed and evaluating photovoltaic system performance [74]. This underscores the importance of control techniques for remote, standalone applications.

## 4 Energy storage technologies

**Fig. 7:**
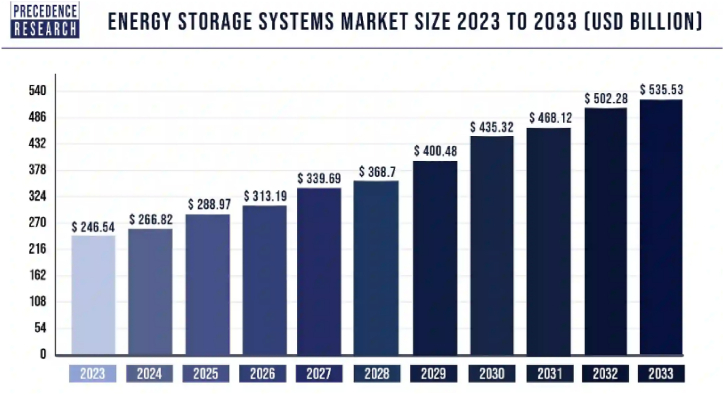
The projected market size and growth of Energy Storage Systems [75].

Fig. 7 shows the projected market size for energy storage systems (ESS) from 2023 to 2033 in USD billion. It shows a steady and significant increase in market size over the decade, starting at $246 billion in 2023 and reaching $535 billion by 2033. This exemplifies the increase in demand for ESS, because of recent breakthroughs in electric vehicle technology and the global trend towards cleaner energy options.

**Fig. 8:**
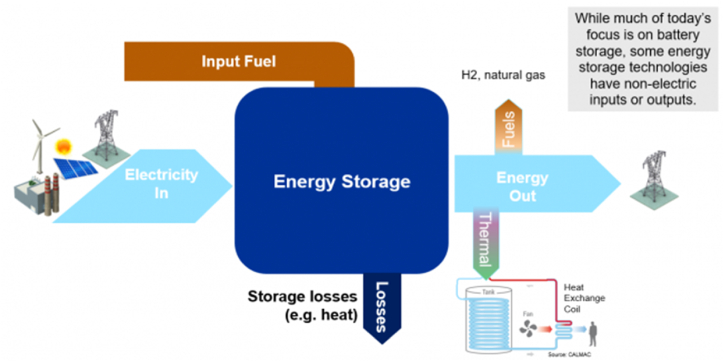
Schemetic of an exemplary hybrid energy storage system.

Based on recent projections, energy storage demands are expected to triple by 2030 [76]. The increased demand is one of the key motivating factors for scientists to develop new ESS that accurately and consistently manage electricity as needed. ESS may be categorized according to several factors, including the type of energy stored, the intended uses, the length of storage, and efficiency. The classification of ESS based on the type of stored energy is used in this article. As Fig. 8 illustrates, ESS can take on a hybrid form that combines two or more different energy storage technologies. An overview of all the energy storage systems and their corresponding forms is presented in Table 2.

**Table 2:** Energy storage systems based on the form of energy.

Energy Storage Systems	Form of Energy
Chemical Energy Storage (CES) System	Hydrogen energy storage
	Synthetic natural gas (SNG) Storage
	Solar fuel
Electrochemical Energy Storage (ECES) System	Battery energy storage (BES)
	Flow battery energy storage (FBES)
	Paper battery
	Flexible battery
Electrical Energy Storage (EES) System	Electrostatic energy storage
	Magnetic energy storage
Mechanical Energy Storage (MES) System	Pumped hydro energy storage (PHES)
	Gravity energy storage (GES)
	Compressed air energy storage (CAES)
	Flywheel energy storage (FES)
Thermal Energy Storage (TES)	Sensible heat storage (SHS)
	Latent heat storage (LHS) or phase change materials (PCM)
	Thermochemical energy storage (TCES)
	Pumped thermal energy storage (PTES)
Others	Hybrid energy storage

### 4.1 Chemical energy storage (CES) System

Chemical energy storage devices use the atomic and molecular bonds of various substances to store energy from chemical reactions [77]. The two most widely used clean energy sources are synthetic natural gas and hydrogen [78]. Coal is used to produce synthetic natural gas (SNG), which is an alternative to conventional natural gas for the generation of power [79]. Thermal gasification turns dry biomass or coal into SNG. Methanation, drying, gasification, purification, and improvement of gas are some of these processes [80]. Tanks or subterranean tunnels are used to store the resultant SNG. Systems for storing hydrogen energy that do not harm the environment produce hydrogen by photocatalytic water splitting or electrolysis. Usually, these systems are made up of an electrolyzer that creates hydrogen, a storage system that holds the hydrogen, and a fuel cell-based conversion unit that uses the stored hydrogen to create electricity as needed [81]. Additionally, researchers have investigated developments in underground hydrogen storage systems and solid-state hydrogen storage. Through their ability to convert sunlight into chemical fuels, solar fuels are essential for the storage of solar energy. To create solar fuels from water and carbon dioxide, scientists are researching thermochemical production processes, artificial photosynthesis, and natural photosynthesis producing electricity, these molecules go through a process where they first become mechanical energy and then electrical energy [82].

### 4.2 Electrochemical energy storage (ECES) System

Flow Battery Energy Storage (FBES) and Battery Energy Storage (BES) are the two primary types of electrochemical energy storage (ECES) systems. FBES is stores energy in a fluid state, typically in tanks or reservoirs, before transferring it to the electrodes [83,84]. This fluid, known as the electrolyte, flows through the battery during charge and discharge cycles. Extended cycle life, scalability, and the flexibility to decouple both energy and power capacity are some of the benefits gained by the implementation of FBES. Common types of FBES include Polysulfide Bromide (PSB) batteries, Vanadium Redox Batteries (VRBs), and Zinc-bromine (ZnBr) batteries. These systems utilize microporous membranes to separate the electrolytes and enable the generation of current.

**Fig. 9:**
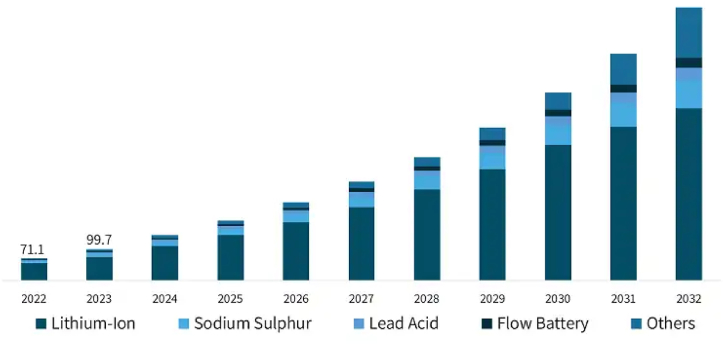
Electrochemical energy storage market share projection 2022–2032 [94].

On the other hand, BES directly stores energy within the electrodes. It involves the transformation of chemical energy into electrical energy through electrochemical reactions. In most cases, BES devices are made up of an electrolyte and an electrode, which acts as a medium that enables the passage of ions between the electrodes [77]. Fig. 9 shows significant growth in the overall BES market over the next decade, with a notable dominance of Lithium-ion (Li-ion) batteries and its utilization and adoption will continue to rise sharply. While lead-acid batteries still play a role in certain applications, their market share is projected to decline as more efficient and advanced battery technologies take precedence. The growth of BES technologies shows the importance of continued innovation and investment in battery technology to meet evolving energy storage needs. Different types of BES exist to cater for diverse applications and requirements. In numerous applications over the years, lead-acid batteries [85] have been utilized extensively, like automotive starting, backup power, and renewable energy storage. Lithium-ion batteries [86,87] are the most widely deployed due to their lightweight design, improved energy density, and longer cycle life. Other types of BES include Solid-State Batteries (SSDs) [88,89], Sodium-ion (Na-ion) [90,91], Nickel-Cadmium (Ni-Cd) [92], and Sodium-Sulphur (NaS) [93].

In the pursuit of lightweight, flexible, and compact energy storage solutions, paper batteries have emerged as an innovative option [95]. These batteries utilized paper as a substrate and incorporated carbon nanotubes to create flexible electrodes. Paper batteries offer advantages such as flexibility, smaller dimensions, and the potential for integration into flexible electronic devices. They operate by generating voltage between two electrodes coated with substances that have opposite electrochemical potentials [96]. Additionally, the use of solid-state electrolytes in flexible batteries enhances safety by minimizing the risk of leaks. Metal-air batteries are another class of energy storage systems that rely on atmospheric oxygen as a key component. These batteries exhibit high energy density, making them attractive for applications requiring long durations of energy supply [97,98].

### 4.3 Mechanical Energy Storage (MES) System

Mechanical Energy Storage (MES) systems are technologies that enable the conversion of energy between mechanical and electrical forms [99]. These systems are vital for managing fluctuations in energy demand and ensuring a reliable and efficient energy supply. Flywheel Energy Storage (FES) is one such device that uses the spinning of a flywheel to store energy as kinetic energy. The flywheel is accelerated by electricity during charging, storing energy in its circular motion. The flywheel slows down and releases its stored kinetic energy, which is then transformed back into electrical power as needed [100]. Another MES technology is Gravity Energy Storage (GES), which utilizes the potential energy of elevated heavy objects. These objects are raised using water and then released to generate electricity as they descend. GES systems consist of components such as pistons, pumps, turbines, and generators [101]. Compressed air energy storage (CAES) devices store energy by compressing and holding onto compressed air in pressurized containers or underground caverns. When the need for power increases, compressed air is released to power turbines, generating energy [102,103].

The most widely deployed MES technology is pumped hydro energy storage (PHES), which involves pumping water to an upper reservoir when surplus electricity is available because demand for electricity is low. The water is released downward, flowing through turbines to produce energy when demand for electricity increases [104]. These MES technologies provide fast response times, allowing for rapid energy conversion when needed. MES systems are known for their high energy efficiency and long cycle lives, making them valuable assets for a resilient and sustainable energy infrastructure.

### 4.4 Electrical Energy Storage (EES) System

Electrical Energy Storage (EES) systems are a critical component of modern energy infrastructure, enabling the efficient storage and utilization of electrical energy. These systems are essential for managing peak demand, grid stability, intermittent renewable energy sources, and overall energy system optimization. Capacitors are a prevalent component in electrostatic systems, storing energy for short-term power distribution across various applications. They sandwich two conductive plates across a non-conductive dielectric substance to create an electrostatic field that stores electrical energy [105]. Capacitors offer rapid charging and discharging capabilities, making them suitable for applications that require high power outputs and quick response times. They find extensive use in electronics, electric vehicles, renewable energy systems, and power electronics. Supercapacitors are an advanced form of electrostatic energy storage. They have higher energy densities compared to traditional capacitors, bridging the gap between capacitors and batteries. Supercapacitors store energy by creating an electrostatic double layer at the interface between the electrode and electrolyte [104,106]. This mechanism allows for high power density, fast charging and discharging rates, and an extended cycle life. Supercapacitors find applications in hybrid vehicles, industrial systems, regenerative braking, and energy-intensive applications that require frequent and rapid energy release.

Superconducting Magnetic Energy Storage (SMES) use the magnetic field produced by a continuous current passing through a superconducting coil to store electrical energy. Permanent magnetic fields are created without any losses thanks to superconductors, which are materials that show zero electrical resistance at low temperatures [107]. SMES systems provide great efficiency, quick reaction times, and a high power density. They are especially well suited for applications like frequency control, backup power systems, and grid stability that need immediate power supply.

### 4.5 Thermal Energy Storage (TES)

Thermal Energy Storage (TES) systems store energy as heat for later use. They employ various processes, including cooling, heating, or phase transitions of substances, to store and release heat energy as required [108]. Sensible Heat Storage (SHS) systems, comprise a range of technologies suitable for diverse temperature ranges and applications [109]. Aquifer Thermal Energy Storage (ATES) is an example of SHS that utilizes the thermal properties of groundwater [110]. During periods of excess or low-cost energy, water is injected into an underground aquifer, transferring thermal energy to the water for later retrieval. ATES systems are commonly used for heating and cooling in large-scale buildings, district energy systems, and industrial processes [111]. Borehole Thermal Energy Storage (BTES) is another form of SHS that involves drilling boreholes into the ground and circulating a heat transfer fluid through a closed-loop system [112]. This allows for the storage and retrieval of heat energy from the subsurface, providing heating and cooling for individual buildings or small-scale applications [109,113]. Molten Salt Thermal Energy Storage (MSTES) is employed for high-temperature applications [114]. In this system, a molten salt, such as a mixture of sodium nitrate and potassium nitrate, is used as the heat transfer fluid. The salt is heated to a high temperature using excess or low-cost energy, and the stored heat is later used for electricity generation or industrial processes requiring high-temperature heat [115]. Hot Water Thermal Energy Storage (HWTES) systems store heat energy in large, insulated tanks filled with hot water. These systems are commonly used in district heating and cooling applications and industrial processes that require a constant hot water supply [116].

In addition to SHS, another type of TES system is latent heat storage (LHS), where heat energy is stored and released through phase transitions of substances. Phase change materials (PCMs) are used for LHS, as they absorb and release large amounts of energy during their phase transitions. Common PCMs include paraffin wax, salt hydrates, and eutectic mixtures [117]. TES systems provide various advantages, including the capacity to shift energy usage to off-peak hours, lower peak demand on the grid, and improve renewable energy efficiency by storing excess energy for later use. They also contribute to improved energy management, reduced energy costs, and increased overall system reliability [[118], [119], [120]]. TES systems, including Sensible Heat Storage and Latent Heat Storage technologies, provide efficient and versatile solutions for thermal energy management. These systems have applications in space heating and cooling, hot water production, industrial processes, and electricity generation.

### 4.6 Hybrid Energy Storage (HES) systems

The underlying principle of Hybrid Energy Storage (HES) systems is to combine the attributes of various ESS to achieve the desired performance. High-power storage systems, which are recognized for their quick response in supplying energy over shorter times, and high-energy storage systems, which react more slowly but supply power over longer times, are the two main categories [121,122]. Pumped Thermal Energy Storage (PTES) and Adiabatic Compressed Air Energy Storage (A-CAES) are two power-density solutions that have recently received a lot of attention from academics [123]. This is a common way to build HES systems by balancing two complementary storage systems, one with a high energy density and the other with a high power density.

## 5 Optimization methods

Optimization is crucial to energy systems' efficiency improvement, cost reduction, and resource maximization, particularly in the context of renewable energy sources. The key objectives of optimization in this domain include maximizing the utilization of renewable resources, minimizing costs, improving system reliability and life span, and reducing carbon emissions. The integration of an ESS is a crucial aspect of HRES, as optimizing the sizing and operation of these energy storage components can further enhance the efficiency, reliability, and flexibility of the overall system. By storing excess energy generated from renewable sources, the ESS can help mitigate the intermittency inherent in renewable energy and improve the system's ability to meet energy demands. Additionally, optimization contributes to grid stability, reliability, and the integration of renewable sources, fostering technological innovation for more sustainable and efficient energy systems.

**Fig. 10:**
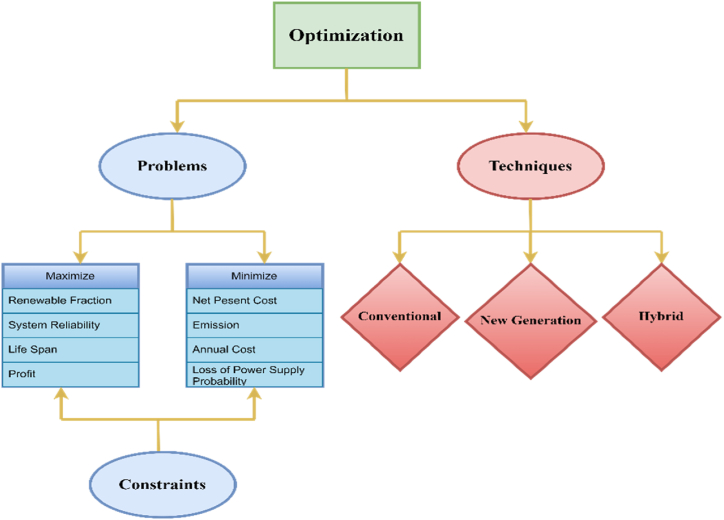
Schemetic of optimization problems, constraints and techniques.

Fig. 10 illustrates the optimization process which involves addressing various “Problems” by applying different “Techniques”. The problems are divided into those where the objective function is maximized and those where it is minimized. To effectively address these problems, optimization techniques are categorized into three, New Generation, Conventional and Hybrid methods. For the optimal sizing of HRES, the Hybrid Solar-Wind System Optimization Sizing (HSWSO) model was developed to optimize the capacity sizes of different components of hybrid solar-wind power generation systems that employ a battery bank. A case study was reported in that paper to show the importance of the HSWSO model for sizing the capacities of wind turbines, PV panels and battery banks of a hybrid solar-wind renewable energy system [124]. Table 3 gives a summary of optimization techniques used in different studies of HRES integrated with ESS.

**Table 3:** Summary of studies considering different HRES integrated with ESS and their chosen optimization techniques and contributions.

Ref	Energy generation	Energy storage	Optimization Techniques	Contribution
[125]	PV, Wind	BESS	Iterative	Minimizes costs while ensuring maximum reliability.
[126]	PV, Wind	BESS	Particle Swarm Optimisation (PSO)	Minimizes the unmet load, emissions, and total cost of the system.
[127]	PV, Wind	BESS + H2-FC Flywheels PHS	PSO, Grasshopper Optimisation Algorithm (GOA)	Assessiesthe reliability of the optimal system configuration in case of primary resource failure.
[128]	PV, Wind	BESS	Annealing PSO	Minimizes operation costs of the integrated system.
[129]	PV, Wind	Hydrogen and BESS	PSO	The feasibility analysis of H_2_ and Battery Power-to-Power (P2P) systems for 21 small islands of France, focusing on techno-economic aspects.
[130]	PV, Wind	BESS + EFCS	PSO	Evaluates a multi-objective optimization problem that considers operating costs, efficiency, and device lifetime to determine the power of energy storage devices.
[131]	PV, Wind	EFCS	PSO	Minimizes CO_2_ emissions, total cost and fuel usage.
[132]	PV, Wind	BESS + EFCS	Genetic Algorithm (GA), Non-dominated Sorting Genetic Algorithm (NSGA)	Minimizes LCOE and unmet load.

### 5.1 New generation optimization methods

New Generation Optimization includes genetic algorithm (GA), particle swarm optimization (PSO), fuzzy logic and neural network algorithms among others. The classification contains strategies often known as metaheuristic optimization methods. GA employs heredity, mutation, crossover, and selection to mimic natural selection [133,134], and operates as a search method. Ammari et al. [135], optimized a hybrid system, including a diesel generator, storage system, wind turbine, and solar generator, to power remote areas of Senegal using a genetic algorithm. Their objective was to reduce CO_2_ emissions and lower the system's levelized cost, establishing an inverse correlation between the two variables.

Fuzzy logic, a mathematical theory grounded in fuzzy sets addresses reality, employing a digital processor to embody human expert knowledge [136]. This was utilized in a study [137] to effectively regulate the energy flow of a hybrid system comprising a battery, wind turbine, and solar photovoltaic, ensuring accurate tracking of imposed input power states. The findings confirmed that the electronic switch signals efficiently monitored the hybrid power system's-imposed input power states. The PSO technique mimics the motion of fish or birds, leveraging their movements in three-dimensional space [138] with each movement standin for a solution. It is seen as the most used method in the new generation optimization methods category. Another study [126] used the PSO algorithm to solve the multi-objective problem optimization problem of the HRES system that includes batteries. Neural network algorithms are trained to perform essential tasks and mimic the structure of the human brain. Amirtharaj et al. [139] used an artificial neural network to determine optimal utilization and minimize switching loss in a system that exhibited superior performance compared to previous optimization techniques.

### 5.2 Conventional optimization methods

Conventional Optimization uses a differential calculation to obtain the optimum solution [140]. Researchers seldom use this method because of their limited space optimization. Numerous conventional optimization methods exist such as iterative, probabilistic, graphical and deterministic. A key idea in conventional optimization methods is the iterative approach, which uses looping structures to repeatedly produce the intended result through a series of phases [141]. In hybrid renewable energy, this approach was used to simulate and optimize systems [142]. Nevertheless, because several loops are required to solve for the maximum and minimum number of components, iterative techniques are time-consuming.

Iterative techniques encompass several methodologies such as linear programming techniques [143,144], dynamic programming techniques [145,146], and multi-objective optimization strategies [[147], [148], [149]]. Probabilistic techniques are applied to handle long-term weather variables, non-linear system element response characteristics, and multi-objective functions. To improve the sizing of hybrid solar-wind generation systems and estimate the long-term average performance, a new probabilistic method was used [150].

To evaluate the probability distribution of power generated from a specified system installed in the South China Sea and optimize an off-grid hybrid energy system, a probabilistic approach was devised. Using the battery level coefficient mode, the method assesses the hybrid system's battery capacity [151]. Optimization programming problems involving two variables are made simpler to solve using graphical methods [152], although different approximations could be needed throughout. Both mathematical and visual methods have their uses in real-world scenarios, yet they are not effective in solving complex issues. Deterministic approaches operate by treating power consumption and energy supply as deterministic parameters with a known time-series variation [153].

### 5.3 Hybrid methods

This approach uses several iterations of the previously described methods. Some of the variations include the parametric approaches, design space approach, quasi-Newton algorithm, response surface methodology, the "Energy hub" concept, matrix approach, the HoneyBee Mating algorithm, the Artificial Immune System algorithm, the Bacterial Foraging algorithm, and tabu search [154]. Several novel metaheuristic algorithms, such as Cuckoo Search, MB Mine Blast, Imperialist Competition, Water Cycle, Hybrid Big Bang-Big Crunch [155] and Covariance Matrix Adaption–Evolution Strategy algorithms, employ PSO techniques as a benchmark for comparison [156]. Feasible deviations arise as a result of combining traditional and new-generation approach methodologies.

An integrated approach for artificial neural networks (ANN) and genetic algorithms (GA) was proposed by Kalogirou [157] to optimize a solar industrial-process heat system, the optimization procedure involved the utilization of the Group Method of Data Handling (GMDH), also known as "polynomial networks". Another study [158] employed GA and PSO to optimize a hybrid system combining solar and concentrator solar photovoltaic with a battery. This system displayed enhanced technical and economic parameters compared to alternative systems and strategies.

### 5.4 Comparison of modelling methodology

A key drawback of optimisation techniques is their complexity and computational time. When it comes to economic optimization, conventional optimization methods are the most used in the literature [159], due to their simplicity and ease of implementation. These methods typically work within a limited parameter space, making them effective for problems where the solution landscape is not highly complex however they are unable to handle the intricate and multi-dimensional nature of integrating HRES. When considering the sizing of high-resolution energy systems, it is commonly recommended to employ analytical methodologies. Simulation approaches that rely on Monte Carlo Simulations (MCS) are deemed unsuitable due to their intricate nature, reliance on specific data that are not always accessible, and the significant increase in processing time. Furthermore, these systems fail to provide users with the ability to discern the essential control variables and the relationships that exist between them [160]. To summarize the most recent optimization techniques used for HRES-EES systems, we have compiled Table 4 to highlight the HRES configuration (energy generation and energy storage type), optimization method and categorization, objectives functions, constraints, and the contribution of the specific literature reviewed. This table shows the similarities and differences in optimization problem formulation for HRES-EES configurations.

**Table 4:** Summary of optimization techniques, objective functions, and constraints in an integrated HRES and ESS model for an energy system.

Ref	Energy generation	Energy storage	Optimization method	Category: New Generation or Conventional or Hybrid	Objective function	Constraint	Contribution
[125]	PV, Wind	BESS	Iterative	Conventional	$P_{G}^{\left(i,j\right)}\left(t\right)=N_{PV}^{i}P_{PV}\left(t\right){PV}_{status}^{i}\left(t\right)+N_{WT}^{i}P_{WT}\left(t\right){WT}_{status}^{j}\left(t\right)\forall i\in \left[1,i_{\max}\right],j\in \left[1,j_{\max}\right],t>0$	Capacity constraint.	Incur minimum costs while ensuring maximum reliability.
						$N_{PV}^{\min}\leq N_{PV}^{i}\leq N_{PV}^{\max}$	
						$N_{WT}^{\min}\leq N_{WT}^{i}\leq N_{WT}^{\max}$	
[126]	PV, Wind	BESS	PSO	New Generation	$Cost=\sum_{j}\left[C_{I,j}+C_{O\&M,j}\times \frac{1}{CRF\left(i,T\right)}+C_{rep,j}\times K_{j}\right]\times P_{j}+C_{fuel}\times fuel_{cons,yr}\times \frac{1}{CRF\left(i,T\right)}$	Constraint of CO_2_ emission	Minimizing the unmet load, fuel emissions, and total cost of the system.
						$C{O_{2}}_{emission}\leq \varepsilon_{CO_{2}}$	
						Constraint of LLP	
						$LLP\leq \varepsilon_{LLP}$	
[127]	PV, Wind	BESS + H2-FC Flywheels PHS	PSO, GOA	Hybrid	$J=\min\;\left(f_{1},f_{2}\right)$ Where: $f_{1}$ is the cost analysis objective function and $f_{2}$ is the environmental objective function.	Constraint of HMG resources $\sum_{t=1}^{8640}\left(P_{PV}\left(t\right)+P_{W}\left(t\right)+P_{dis}\left(t\right)\right)\geq \sum_{t=1}^{8640}\frac{E_{l}\left(t\right)}{\eta_{inv}}$	The study introduced hybrid optimization to enhance the optimization process for HRES. It minimized both economic (LCOE) and environmental impact (greenhouse gas emission).
						Constraints of HBSS energy management	
						$P_{HBSS}\left(t\right)=P_{HBSS}\left(t-1\right)+P_{g}\left(t\right);P_{g}\left(t\right)\geq 0$	
[128]	PV, Wind	BESS Hydrogen	Annealing PSO	Hybrid	$C_{day}=\sum_{t=t_{0}}^{t_{end}}C_{ele}P_{ele}^{t}\Delta t+\sum_{t=t_{0}}^{t_{end}}C_{grid}P_{grid}^{t}\Delta t+\sum_{t=t_{0}}^{t_{end}}C_{sto}P_{bat}^{t}\Delta t+\sum_{t=t_{0}}^{t_{end}}C_{com}E_{H_{2}}^{t}\Delta t$	System power balance constraint	Making the operation of the integrated system more cost-efficient
						$P_{renew}^{t}+P_{bat}^{t}+P_{grid}^{t}=P_{ele}^{t},\forall t\in \left[t_{start},t_{end}\right]$	
						BESS Constraint	
						$S_{bat}^{t}=S_{bat}^{t-1}\left(1-\sigma \right)-\eta_{c}\frac{P_{bat}^{t}\Delta t}{E_{bat}}$	
						Grid Interactive power constraint	
						$P_{grid}^{\min}\leq P_{grid}^{t}\leq P_{grid}^{\max},\forall t\in \left[t_{start},t_{end}\right]$	
						Electrolyser Operation Power Constraint.	
						$P_{ele}^{\min}\leq P_{ele}^{t}\leq P_{ele}^{\max},\forall t\in \left[t_{start},t_{end}\right]$	
[129]	PV, Wind	Hydrogen and BESS	PSO	New Generation	$cost=\sum_{j}\left[C_{I,j}+C_{O\&M,j}\times \frac{1}{CRF\left(i,T+\right)}+C_{rep,j}\times K_{j}\right]\times P_{j}+C_{fuel}\times {fuel}_{cons,yr}\times \frac{1}{CRF\left(i,T+\right)}$	CO_2_ emission and LLP during one year	The feasibility analysis of H_2_ and Battery based Power-to-Power (P2P) systems for 21 small islands of France, focusing on techno-economic aspects.
						$LLP\leq \varepsilon_{LLP}$	
						${CO}_{2_{emission}}\leq \varepsilon_{{CO}_{2}}$	
[130]	PV, Wind	BESS + EFCS	PSO	New Generation	The single-objective optimization $\min\left\{OF=f(P_{bat},P_{fc,}P_{lz}\right\}$ The multi-objective optimization $MOF\left(x\right)=\frac{w_{1}}{f_{1}^{\max}}\cdot \left[C_{bat}\cdot P_{bat}+C_{fc}\cdot P_{fc}+C_{lz}\cdot P_{lz}\right]+\frac{w_{2}}{f_{2}^{\max}}\cdot \left[1-\frac{P_{bat}+P_{fc+}P_{lz}}{P_{bat}^{in}\left(P_{bat}\right)+P_{fc}^{in}\left(P_{fc}\right)+P_{lz}^{in}\left(P_{lz}\right)}\right]+\frac{w_{3}}{f_{3}^{\max}}\cdot \left[\frac{0.5\cdot P_{bat\cdot \Delta t}}{E_{bat}^{nom}\cdot N_{cycle}^{\max}\cdot B\left(P_{bat}\right)}+\frac{H_{fc}^{sto}+{Li}_{fc}\left(P_{fc}\right)\cdot \left(3-H_{fc}^{sto}\right)}{H_{fc}^{\max}}+\frac{H_{lz}^{sto}+{Li}_{lz}\left(P_{lz}\right)\cdot \left(3-H_{lz}^{sto}\right)}{H_{lz}^{\max}}\right]$	$\begin{array}{l} 0\;<P_{bat}\;<\;P_{bat,dis}^{\max}\\ 0<P_{fc}<P_{fc}^{\max}\\ P_{lz}=0\\ P_{bat}+P_{fc}=P_{net} \end{array}\}\;with\;P_{net}\geq 0$	The study involves evaluating a multi-objective optimization problem that considers operating costs, efficiency, and device lifetime to determine the power of energy storage devices.
						$\begin{array}{l} 0<P_{bat}<P_{bat,char}^{\max}\\ P_{fc}=0\\ 0<P_{lz}<P_{lz}^{\max}-P_{bat}-P_{lz}=P_{net} \end{array}\}\;with\;P_{net}<0$	
[131]	PV, Wind	EFCS	PSO	New Generation	Total net present cost $\min_{N_{DER}}TNPC={NPC}_{PV}+{NPC}_{WT}+{NPC}_{FC}+{NPC}_{EL}+{NPC}_{HSS}+{NPC}_{SMR}+{NPC}_{Emission}+{NPC}_{Fuel}+{NPC}_{Buy}-{NPC}_{Sell}$	$P_{{DER}_{i}}^{\min}\leq P_{{DER}_{i}}\left(t\right)\leq P_{{DER}_{i}}^{\max}$	Reduce the CO_2_ emissions, total cost and fuel usage.
						$P_{PV}\left(t\right)\times \eta_{DC/DC}+P_{WT}\left(t\right)\times \eta_{AC/DC}+P_{FC}\left(t\right)\times \eta_{DC/AC}=P_{load}\left(t\right)-\xi_{Buy}\left(t\right)\times P_{{Buy}_{grid}}\left(t\right)+\xi_{Sell}\left(t\right)\times P_{{Sell}_{grid}}\left(t\right)$	
[132]	PV, Wind	BESS + EFCS	GA, NSGA-II	Hybrid	$fit=\left({fit}_{r}\prod_{i=1}^{n_{c}}\right)\left(1-{fit}_{0}\right)+{fit}_{0}$	$Y_{3}\leq Y_{3,c}$	The study reduces LCOE and unmet load.
						$Y_{4}\leq Y_{4,c}$	
						$X_{l}\leq X\leq X_{u}$	
[161]	PV, Wind	BESS Hydrogen tanks	GA, NSGA-II	Hybrid	$Minimize\;ATC\left(N_{pv},N_{wt},N_{bat},N_{ht},N_{fc},N_{el}\right)$	PWB	The study minimized the annual total cost.
						$0\leq N_{pv}\leq N_{pv,\max}$	
						$0\leq N_{wt}\leq N_{wt,\max}$	
						$0\leq N_{bat}\leq N_{bat,\max}$	
						$E_{bat,\min}\leq E_{bat}\left(t\right)\leq E_{bat,\max}$	
						PWF	
						$0\leq N_{pv}\leq N_{pv,\max}$	
						$0\leq N_{wt}\leq N_{wt,\max}$	
						$0\leq N_{ht}\leq N_{ht,\max}$	
						${HST}_{\min}\leq HST\left(t\right)\leq {HST}_{\max}$	
[162]	PV, Wind	Hydrogen storage	Firefly Shuffled frog leaping algorithm PSO	Hybrid	$\min.NPC\left(N_{PV},N_{ELZ},N_{WT},N_{FC},N_{tank}\right)$	$N_{PV}^{\min}\leq N_{PV}\leq N_{PV}^{\max}$	The NPC is reduced with a LPSP of 2%.
						$N_{bat}^{\min}\leq N_{bat}\leq N_{bat}^{\max}$	
						$N_{WT}^{\min}\leq N_{WT}\leq N_{WT}^{\max}$	
						$N_{FC}^{\min}\leq N_{FC}\leq N_{FC}^{\max}$	
						$N_{tank}^{\min}\leq N_{tank}\leq N_{tank}^{\max}$	
[163]	PV, Wind, Biomass,	Fuel cell Hydrogen tank Battery bank	GA	New Generation	TotalNetPresentCost(TNPC)=CC+O&MC+RC+FC	Battery storage constraint	Reduce TNPC, COE, unmet load and CO_2._
						$E_{BATT\;\min}\leq E_{BATT}\left(t\right)\leq E_{BATT\;\max}$	
						Bounds constraint	
						$N_{WTG}=Integer,0\leq N_{WTG}\leq N^{\max_{WTG}}$	
						$N_{SPV}=Integer,0\leq N_{SPV}\leq N^{\max_{SPV}}$	
						$N_{Batt}=Integer,0\leq N_{Batt}\leq N^{\max_{Batt}}$	
						Power reliability constraint	
						UnmetLoad=(yearlyloadsupplied/totalload)	
[164]	PV, Wind, Biomass	Hydrogen Storage, BESS	P-graph	Conventional		Energy supply and demand constraints	Minimize emissions and cost.
						$\sum_{j}C_{j,t}^{n}\geq \sum_{j}{Generation}_{j,t}^{n}\geq {Demand}_{t}^{n},\forall n\in N,t\in T$	
						$C_{j,t}^{n}={\mathrm{Max}\;generation}_{j,t}^{n}$	
						Energy balance constraints	
						$C_{t}^{ele}+C_{t}^{heat}\geq {Gen}_{t}^{SCP,ele}+{Gen}_{t}^{BCHP,ele}+{Gen}_{t}^{HG,ele}+{Gen}_{t}^{WDG,ele}+{Gen}_{t}^{SCP,heat}+{Gen}_{t}^{BCHP,heat}+{Gen}_{t}^{BF,heat}\geq {Demand}_{t}^{ele}+{Demand}_{t}^{heat}$	
[165]	PV, Biomass	BESS	Iterative	Conventional	$Minimize:TC=\sum_{d=1}^{d_{n}}\sum_{j=1}^{6}\sum_{t=1}^{24}\left[C_{j}\times E_{jdt}\right]$	Energy balance constraint	Minimize cost.
						$E_{jj=BATT}\left(t\right)\pm E_{BATT}\left(t\right)\geq E_{Load}\left(t\right)+E_{Dump}\left(t\right)$	
						Individual capacity constraint	
						$P_{j}\left(t\right)=Cap_{j}\;for\;all\;t$	
						Unit generation limits	
						$P_{j\;\min}\leq P_{j}\left(t\right)\leq P_{j\;\max}\;for\;all\;t$	
						Battery storage limit	
						$E_{BATT\;\min}\leq E_{BATT}\left(t\right)\leq E_{BATT\;\max}\;for\;all\;t$	
						$E_{BATT\;\min}=\left(1-DOD\right)E_{BATT\;\max}$	
[166]	PV, Wind	Hydrogen storage	GA	New Generation	$\underset{x}{\to }=\mathrm{argmin}\left[LCOE\left(\underset{x}{\to }\right)\right]$	Constraints on design parameters	Minimize cost.
						$1\leq N_{nuclear}\leq 80$	
						$5000\leq N_{wind}\leq 15000$	
						$100\leq A_{solar}\leq 1000{km}^{2}$	
						Total energy generated exceeds the energy demand at all time	
						$E_{gen}\left(t\right)-D\left(t\right)\geq 0\forall t\in \left[t_{0},t_{f}\right]$	
[167]	PV, Wind	Hydrogen storage	Flower pollination algorithm	Conventional	$minimizeTNPC=NPC_{comps}+NPC_{Load-loss}+Penalty$	Loss of load expected	Minimize TNPC Reliability of LOLE and LOEE.
						$LOLE=\sum_{t=1}^{N}E\left(LOL\left(t\right)\right)$	
						Loss of energy expected	
						$LOEE=EENS=\sum_{t=1}^{N}E\left(LOE\left(t\right)\right)$	
[168]	PV, Wind	Hydrogen Electrochemical	NSGA-II	New Generation	$\min_{x}F\left(X\right)=\left[TCE\left(X\right),LCOE\left(X\right)\right]$	Wind farm constraint	Multi-objective optimization is used to coordinate total CO_2_ emission and LCOE.
						$s.t.\;P_{wt}^{\left(L\right)}\leq P_{wt}^{in}\leq P_{wt}^{\left(U\right)}$	
						PV plant constraint	
						$P_{PV}^{\left(L\right)}\leq P_{PV}^{in}\leq P_{PV}^{\left(U\right)}$	
						Electrolyzer constraint	
						$P_{ele}^{\left(L\right)}\leq P_{ele}^{in}\leq P_{ele}^{\left(U\right)}$	
						Battery bank constraint	
						$E_{batt}^{\left(L\right)}\leq E_{batt}^{in}\leq E_{batt}^{\left(U\right)}$	
						The power output of the total system constraint	
						$E_{total}\left(t\right)\geq E_{load}\left(t\right)$	
[169]	PV, Wind	BESS EFCS	PSO GA	Hybrid	$minimize:waCOE=\frac{P_{RES}\times CosE+P_{Grid}\times GT}{D}$	$F_{RES}<F_{RES,\min}$	Minimize the cost of energy.
[170]	PV, Wind, Biomass	BESS	PSO Harmony search Jaya algorithm	Hybrid	$MinimizeC_{NPC}\left\{N_{PV},N_{bat},N_{gen},N_{WT}\right\}$	$N_{PV}^{\min}\leq N_{PV}\leq N_{PV}^{\max}$	To establish an optimal combination of the system components to attain a minimum value of the net present cost of the system.
						$N_{bat}^{\min}\leq N_{bat}\leq N_{bat}^{\max}$	
						$N_{gen}^{\min}\leq N_{gen}\leq N_{gen}^{\max}$	
						$N_{WT}^{\min}\leq N_{WT}\leq N_{WT}^{\max}$	
						$0\leq LPSP\leq {LPSP}_{\max}$	
						$0\leq EEF\leq {EEF}_{\max}$	
[171]	PV, Wind	Hydrogen tank	Simulated annealing Harmony search Chaotic simulated annealing Simulated annealing-based harmony search Chaotic simulated annealing-based harmony search	Hybrid	$Minimize\;LCC\left(A_{WT},A_{PV},N_{HT}\right)=\sum_{m\in WT,PV,HT,FC,Ele,Conv/Inv}{CC}_{npv,m}$	$A_{WT}\geq 0$	Minimize total cost.
						$A_{PV}\geq 0$	
						$N_{HT}\geq 0$	
						${LPSP}^{m}\geq LPSP$	
[172]	PV, Wind	BESS	NSGA-II PSO Harmony search	Hybrid	$Minimize\;Z=\sum_{n=pv,wt,bat,ro,inv}TAC_{n}+{\left(m\left(\geq 10^{6}\right)*\left(LPSP-{LPSP}_{\max}\right)\right)}^{3}$ $LPSP\leq {LPSP}_{\max}\left(=0,1,2,5\;and\;10\right)\left(\%\right)$	$\left\{ \begin{array}{l} \begin{array}{l} 0\leq N_{pv}\leq N_{pvmax}\\ 0\leq N_{wt}\leq N_{wtmax}\\ 0\leq N_{bat}\leq N_{batmax} \end{array}\\ Q_{pmin}\leq Q_{p}\leq Q_{pmax} \end{array}\right.$	Incur minimum costs while ensuring maximum reliability.
						In battery inclusive instance:	
						$P_{bat.\min}\leq P_{bat}\leq P_{bat.\max}$	

The new generation methods have shown promise in optimizing the integration of HRES and ESS. These methods show high speed, good accuracy, and efficiency, making them suitable for complex optimization problems. Their heuristic nature allows them to quickly find the best solution, which is essential for managing the dynamic and unpredictable behaviour of renewable energy sources and storage systems [173]. One study [174] has suggested that new generational algorithms are the most appropriate approach for optimizing HRES due to their ability to operate independently of long-term meteorological input data. Another study [155] emphasized the heuristic nature and rapid convergence to the global optimum as defining characteristics of new generational algorithms. However, it is important to note that these algorithms experience reduced optimized performance as the number of constraints increases rapidly and without control. Their result is a significant increase in the computational load and the rate at which the algorithms diverge [148].

The hybrid method demonstrates a strategy that is resilient and fast, research has shown that it is the most effective approach for integrating HRES and ESS because it combines the strengths of different optimization techniques to handle the limitations of single-method approaches [175]. For instance, a hybrid approach may utilize PSO for its fast convergence and GA for its robustness creating a comprehensive optimization strategy that balances speed, accuracy, and resilience. Table 4 indicates that hybrid optimization methods is the most common approach for the integration of HRES and ESS. These methods are particularly effective in managing the complexities of multiple energy sources and storage systems. They can handle a diverse set of constraints and objectives, but require intricate design and complex code [155].

## 6 Optimization criteria and constraints

The evaluation of the HRES-EES integrated system's optimization process depends on a variety of constraints or criteria. These include user-selected constraints representing predetermined requirements and relevant objectives [176], sometimes classified as scientific, functional, and practical [177]. The conceptualization of the decision-making process for a renewable energy system project outlined a framework that pinpointed technological, organizational, ecological, financial, and communal barriers [178]. Within the multi-dimensional decision analysis framework, 36 sustainability assessment indicators were categorized into quantitative and qualitative categories. Quantitative indicators predominantly use economic metrics, while qualitative indicators use environmental and social metrics, forming the foundational pillars for sustainability metrics [177].

A sustainability assessment of seven projects emphasized the crucial role of indicator selection in the evaluation process [179]. The development of a universally applicable sustainability indicator for HRES evaluation involved the use of the Analytical Hierarchy Process (AHP), incorporating economic, environmental, and social sub-indicators [180]. In another study seventeen sustainability indicators were classified into environmental, economic, and social categories [181]. A literature survey grouped assessment indicators into three main categories: technical (power reliability), environmental, and economic [176]. Given that the focus of this research is on the reliability and efficiency of the system, this study will concentrate on the power reliability indicator, which is extensively described below.

### 6.1 Power reliability indicators

Previous studies have commonly addressed the optimization challenges by considering technical, financial, and environmental aspects concurrently [182,183]. While numerous power reliability indicators have been identified focusing solely on the technical approach, these indicators serve as comprehensive metrics representing the system's overall behaviour, indicating its performance compliance with required levels for specified time intervals and conditions [184]. The Loss of Load Expectation (LOLE), Loss of Healthy Expectation (LOHE), System Average Interruption Duration Index (SAIDI), Service Quality Index (SQI), Fractional Load Not Served (FLNS), Loss of Energy Expectation (LOEE), Exergetic Capacity Factor (ExCF), Electricity Match Rate (EMR), and Maximum Expected Energy Supplied (maxENS) are examples of these global indicators [185,186]. This study reviews several power reliability indicators commonly featured in scientific journals, such as RPS (Reliability of 10.13039/100028466Power Supply), LOLR (Loss of Load Risk), LOLP (Loss of Load Probability), EENS (Expected Energy Not Supplied), LOLH (Loss of Load Hours), FEE (Final Excess Energy), 10.13039/501100018815ELF (Equivalent Loss Factor), EUE (Expected Unserved Energy), LA (Level of Autonomy), SPL (System Performance Level), EGR (Energy Generation Ratio), EIR (Energy Index of Reliability), and 10.13039/501100001600REF (10.13039/100017275Renewable Energy Fraction), along with LOPSP (Loss Of 10.13039/100028466Power Supply Probability).

Notably, these power reliability measures are categorized as energy loss indicators, except for LOLH, LOLP and LOLR, which are categorized as load loss indicators [187]. Specifically, LOLH measures the number of hours during simulations when the load demand surpasses the power supply from the ESS and energy sources on an hourly basis, excluding component breakdowns or maintenance downtime from the calculation [188,189].

(1) $${LOLH}_{current}\left(hours\right)=\left\{ \begin{array}{l} {LOLH}_{previous}+1,\forall P_{Load}>P_{HRES,\max}\;\begin{array}{cc} \& & {SOC}_{ESS},current \end{array}={SOC}_{\min}\\ {LOLHP}_{previous},otherwise \end{array}\right.$$

In equation (1), $P_{Load}$ represents the load demand, $P_{HRES,\max}$ denotes the highest amount of power generated by each producing unit, ${SOC}_{ESS}$ signifies the current State Of Charge (SOC) of the ESS, and ${SOC}_{\min}$ is the minimum SOC of the ESS. The LOLP is a predictive measure (which can be modelled with equations (2), (3), (4)) indicating the duration for which an energy system's load denoted as L(t), exceeds the generating resources capacity P(t) [190]. LOLP is derived through probability network modeling using a binomial distribution [191], and its expression is articulated as in Refs. [176,188].

(2) $$LOLP\left(\%\right)=\frac{\sum_{t=1}^{T}\varphi \left(t\right)}{T}=\frac{LOLH}{T}$$

Where: $\varphi \left(t\right)=\left\{ \begin{array}{l} 0,for\;L\left(t\right)\leq P\left(t\right)\\ 1,for\;L\;\left(t\right)>P\left(t\right) \end{array}\right.$:

(3) $$LOLP\%=\frac{\int_{0}^{T}Power\;deficit\left(t\right)}{\int_{0}^{T}Power\;demand\;\left(t\right)}$$

(4) $$LOLP\left(\%\right)=\sum_{j}P\left[C_{A}=C_{j}\right]-P\left[L>C_{j}\right]=\sum_{j}\frac{P_{j}*\;t_{j}}{100}$$

where P is the probability, $C_{A}$ as the available generating capacity, ${\;C}_{j}$ as the remaining generating capacity, L as the expected load, $P_{j}$. as the probability of capacity outage, and $t_{j}$ as the percentage of time the expected load L surpasses the remaining generating capacity, ${\;C}_{j}$.

The fourth equation, which permits the use of a load duration curve in LOLP computation, was developed under the supposition that the peak load remains constant throughout the day. Furthermore, LOLP applies solely to generation facilities (Hierarchical Level I), while the Probability of Load Curtailment indicator considers both generation and distribution networks (Hierarchical Level II) [184,192]. SPL, in contrast, tallies the days when the load is not met and derives its values from probabilities generated by Markov chain modeling [193]. LOPSP, a frequently utilized power reliability indicator, indicates the probability of insufficient power supply over a specified duration. This can be represented with Equation (5) [194,195] or Equation (6) [176,196].

(5) $$LOPSP\left(non-dimensional\right)=\frac{\sum_{t=1}^{t=T}hours\;\left(I_{supplied}\left(t\right)<I_{required}\left(t\right)\right)}{T}$$

In this context, the variables are defined as follows: $I_{supplied}\left(t\right)$ represents the current supplied by the generating sources of the HRES at hour t, $I_{required}\left(t\right)$ signifies the current required by the load at hour t, and T denotes the total number of hours.

(6) $$LOPSP\;\left(\%\right)=\frac{\sum_{t=1}^{t=T}LPS\left(t\right)}{\sum_{t=1}^{T}E_{D}\left(t\right)}$$

In equation (6), the variables are defined as follows: T is the length of hours in the evaluation period; LPS(t) is the energy deficit at that period t (the point at which batteries have achieved their Depth of Discharge and energy output from renewable resources is insufficient); and $E_{D}\left(t\right)$ is the energy needed by the load at period t [197]. It is important to emphasize that the time interval in which the load is not satisfied, as a result of inadequate production of energy from renewable sources and the ESS, approaches its maximum DOD at what is referred to as the power failure period. The second method for implementing the LOPSP uses probabilistic methodologies that account for the variable and stochastic characteristics of resources and load, thereby negating the need for time-series simulations [196]. Off-grid HRES tolerance typically falls within the range of 0.05–2% [198,199]. Reliability of power supply (RPS) is sometimes used as a complementary measure to LOPSP and is calculted using equation (7) [200]. Expected Energy Not Supplied (EENS) serves as a probabilistic index, reflecting the anticipated energy demanded by the load but not delivered due to inadequate generation capacity, and is calculated using Equation (8) [176,201].

(7) RPS=1−LOPSP

(8) $$EENS\left(kWh/year\right)=\left\{ \begin{array}{l} E_{Load}-\int_{P_{HRES,\min}}^{P_{HRES,\max}}P_{HRES}.fP_{HRES}\left(P_{HRES}\right).d\left(P_{HRES}\right),for\;P_{Load}>P_{HRES,\max}\\ \int_{P_{HRES,\min}}^{P_{Load}}\left(P_{Load}-P_{HRES}\right).fP_{HRES}\left(P_{HRES}\right).d\left(P_{HRES}\right),for\;P_{HRES,\min}\leq P_{Load}\leq P_{HRES,\max}\\ 0,for\;P_{Load}<P_{HRES,\min} \end{array}\right.$$

In this context, the variables are defined as follows: Convolution of the separate probability density functions (PDFs) for each generating unit in the studied Hybrid Renewable Energy System (HRES) yields $fP_{HRES}$, the PDF for all the units that produce electricity. $P_{Load}$ represents the load demand, $E_{Load}$ is the energy requested by the load, $P_{HRES}$ is the power produced by all generating units, $P_{HRES,\max}$ is the maximum power output from all generating units and $P_{HRES,\min}$ is the minimum power produced by all generating units. The alternative formula for calculating Expected Energy Not Supplied (EENS) in Refs. [202,203], requires a load duration curve. Note that EENS has the lowest convergence rate [192], so it is advised to use it as the convergence target in multi-optimization analyses. Equation (9) is used to calculate the Energy Index of Reliability (EIR) [202,204].

(9) $$EIR\left(non-dimensional\right)=1-\;\frac{EENS}{E_{D}}$$

The load energy demand is represented as $E_{D}$ and for a proportion of time, measured in hours.When load losses are observed the loss area (LA) is calculated using Equation (10) [176,201].

(10) $$LA\left(\%\right)=1-\;\frac{H_{LOL}}{H_{tot}}=1-\;\frac{LOLH}{H_{tot}}$$

In this context, $H_{LOL}$ represents the sum of hours with load losses, and $H_{tot}$ denotes the t for the total hours of operations. ELF represents the hours of outage divided by the total operating hours [205,206] as shown in Equation (11).

(11) $$ELF\;\left(undefined\;representation\right)=\frac{\sum_{t}\frac{q_{h}}{d_{h}}}{H}$$

In the simulation's $h_{th}$ step, the Loss of Load $q_{h}$, power demand $d_{h}$, and the total simulation hours H are calculated. Equation (11) enables ELF to assess both the frequency and extent of power outages [207]. An ELF value below 0.01 is deemed satisfactory for standalone and remote rural setups. According to a study [205], developed countries with grid-connected systems aim for an ELF reliability indicator of 0.0001. During the system evaluation period, we calculate the expected energy that the load does not receive (EUE), a deterministic index theoretically comparable to EENS.EUE is derived from Equation (12) using a clustering algorithm with negative margin probabilities [208].

(12) $$EUE\left(kWh/\;\text{year}\right)=\Delta T\cdot \sum_{k=1}^{N_{t}}U_{k}$$

where $\Delta T=\frac{T}{N_{t}}$ represents the length of the discrete-time step, considering that the observation period T is divided into $N_{t}$ the discrete time steps and $U_{k}$ which represents anticipated unmet loads for “kth” time step. Following the analysis period, FEE is equivalent to the net charge accumulated in ESS. Equation (13) is used to express this indicator as the difference in the ESS charge at the start and finish of the investigation.

(13) $$FEE\;\left(kWh/year\right)=CE_{t=T}-CE_{t=0}$$

where $CE_{t=T}$ represents the ESS average energy at the end of the analysis; $CE_{t=0}$ is the ESS accumulated energy at the beginning, and T indicates the total duration of the analysis period. The Final Excess Energy (FEE) serves as an indicator of the net change in ESS charge. A negative FEE implies a potential decrease in net ESS charge, posing a risk of system failure, while a zero FEE indicates consistency in the initial and final ESS charge. Conversely, a positive FEE suggests an increase in net ESS charge, potentially leading to overestimation. FEE aims to minimize system costs to zero, allowing for a slightly positive number as necessary to counter potential load fluctuations. To maintain energy generation equilibrium in these situations, surplus energy is directed towards a simulated load [186].

Renewable Energy Fraction (REF) is an indicator of power dependability for grid-dependent and off-grid HRES, which consist of both RES and non-intermittent power. Equation (14) can be used to quantify the percentage of load that is met by renewable energy supplies [209].

(14) $$REF\;\left(\%\right)=1-\;\frac{\sum_{t=0}^{t=T}E_{NIS}\left(t\right)}{\sum_{t=0}^{t=T}L\left(t\right)}$$

The REF is a crucial metric for evaluating the adequacy of the hybrid system. Where ∑t=Tt=0L(t) is the cumulative load demand during the time frame and $T,E_{NIS}\left(t\right)$ represents the energy generated by non-intermittent resources. A REF of zero denotes that all non-intermittent energy resources have met the load, whereas a REF larger than one shows that the hybrid system's sizing was overestimated [210]. On the other hand, a REF of less than zero indicates that the system's sizing was underestimated. The effectiveness and efficiency of the hybrid energy system design are influenced by the balance between non-intermittent energy generation and total load demand (Equation (15)), which is determined by REF.

(15) $$EGR=\frac{{GF}_{WE}}{{GF}_{PV}}$$

Equation (16) represents ${GF}_{WE}$, which indicates the percentage of total energy produced that is supplied by wind turbines.

(16) $${GF}_{WE}=\frac{\sum_{t=0}^{t=T}E_{WE}\left(t\right)}{\sum_{t=0}^{t=T}E_{PV}\left(t\right)+\sum_{t=0}^{t=T}E_{WE}\left(t\right)}$$

Equation (17) represents the Gross Fixed Production Value ${GF}_{PV}$, which is the percentage of total energy produced byphotovoltaic (PV) modules.

(17) $${GF}_{PV}=\frac{\sum_{t=0}^{t=T}E_{PV}\left(t\right)}{\sum_{t=0}^{t=T}E_{PV}\left(t\right)+\sum_{t=0}^{t=T}E_{WE}\left(t\right)}$$

Where $E_{WE}\left(t\right)$ is the energy generated by wind turbines at moment t, $E_{PV}\left(t\right)$ is energy generated by photovoltaic (PV) modules at moment t. When modelling or developing a HRES, one of the key variables to consider is the Energy Generation Ratio or EGR. When EGR is equal to 1, it means that wind turbines and PV modules have contributed the same amount to load satisfaction. When wind turbines contribute more than PV modules, the EGR is greater than 1; when the EGR is less than 1, PV modules contribute more [211]. During the feasibility study phase, EGR acts as an optimization target for HRES designers, making it easier to create a system that gives priority to a particular energy resource that is prevalent in the region. It is crucial to consider the challenges of over-sized systems and power loss under uncertainties. Over-sizing the system leads to unnecessary capital expenditure and environmental impact. This can be mitigated by optimizing the Capacity Utilization Factor (CUF) and ensuring that the generation and storage capacities do not excessively exceed the peak demand. Equations (18), (19) represent this constraint:

(18) $$CUF=\frac{Actual\;Energy\;Output}{Installed\;Capacity\times Time\;Period}$$

(19) $$P_{gen}^{\max}+P_{stored}^{\max}\leq \alpha \times P_{demand}^{peak}$$

Moreover, the system must be resilient to uncertainties in renewable energy generation and load demand. This requires stochastic modelling and robust optimization techniques to minimize the risk of power loss. The Probability of Loss of Power (PLP) is a critical metric for this purpose. Other constraints that could be considered are further discussed in the next section.

### 6.2 Environmental constraints

Reducing greenhouse gas emissions of hybrid systems relative to conventional systems is a key environmental constraint [2] and it is represented by Equation (20).

(20) $$\Delta E=E_{conv}-E_{hybrid}$$

Where: ΔE stands for emission reduction, $E_{conv}$ for emissions from conventional energy sources, and $E_{hybrid}$ for emissions from the hybrid system.

Resource efficiency is another contraint which covers the efficient use of natural resources in the installation and operation of HRES and ESS as modelled by Equation (21).

(21) $$\eta_{\text{resources}}=\frac{E_{useful}}{E_{total}}$$

Where: $\eta_{\text{resources}}$ represents Resource efficiency, $E_{useful}$ represents useful energy output, and $E_{total}$ represents total energy input from the resource.

### 6.3 Economic constraints

Capital Expenditure (CapEx) is a key economic constraint which covers the initial cost of installing HRES and ESS [[212], [217]] and is calculated using Equation (23).

(22) $$CapEx=\sum_{i}C_{i}$$

Where $C_{i}$ stands for the cost of component i:

Another constraint in this category is operational Expenditure (OpEx**)** which covers the cost of both operation and maintenance [[213], [217]] and is calculated using Equation (23).

(23) $$OpEx=\sum_{i}\left(C_{m,i}+C_{o,i\;}+C_{r,i}\right)$$

Where $C_{m,i}$ is the maintenance cost of the components ,i, $C_{o,i\;}$ is the operational cost of the components ,i, and $C_{r,i}$ is the replacement cost of the components ,i.

The other constraint considered here is net present value (NPV), which ensures that the overall system is economically viable [214]. It is calculated using Equation (24).

(24) $$NPV=\sum_{t=0}^{T}\frac{R_{t}-C_{t}}{{\left(1+r\;\right)}^{t}}$$

Where: NPV is the net present value, $R_{t}$ is the revenue at time t, $C_{t}$ is the cost at time ,t, r is the discount rate, and T is the time period.

## 7 Conclusion and future work

This review paper explores the integration of EES technologies with HRES and associated optimization techniques. Integrating these systems is crucial for enhancing the reliability and efficiency of renewable energy utilization, as it helps address the intermittency and variability inherent in renewable energy sources. Key takeaways from this review include:

• Hybrid optimization techniques are the most effective approach for integrating HRES and ESS because they combine the strengths of different optimization techniques to handle the limitations of single-method approaches.
• Most studies aimed to maximize system reliability while minimizing costs. This includes ensuring a consistent energy supply and minimizing load loss probabilities, which is critical for user acceptance and system viability.
• Capacity and CO_2_ emissions constraints were frequently considered in studies. Capacity constraints ensure that the number of components like solar panels and wind turbines stays within realistic limits, which is crucial for designing feasible systems. Emission constraints are used to limit the CO_2_ emissions from these HRES. Accounting for both capacity and emission constraints is essential for developing practical and environmentally friendly systems that can be widely adopted.

Implementing HRES faces several technical challenges, including the need for advanced optimization techniques to handle complex system configurations and operational strategies. Additionally, managing the lifetime and efficiency of ESS, especially under varying load conditions, remains a critical area for further research. Yet, adopting HRES in combination with ESS contributes to reducing greenhouse gas emissions and promoting clean energy use.

Future research should focus on developing more sophisticated optimization algorithms that can better handle the uncertainties, stochasticity, and dynamic nature of renewable energy resources. Investigating the feasibility of large-scale deployment of HRES and addressing technical challenges such as system integration, scalability, and maintenance will be crucial. Additionally, exploring policy frameworks and financial models to support the adoption of HRES in various industrial sectors can accelerate the transition to sustainable energy systems.

## Ethical approval

None of the authors conducted studies involving human participants or animals in this article.

## Informed consent

All participants included in the study provided informed consent.

## Data availability statemement

All data referred to throughout the article are presented in the manuscript. No code was used in the development of the article.

## CRediT authorship contribution statement

**Oluwatoyosi Bamisile:** Writing – review & editing, Validation, Supervision, Methodology, Investigation, Conceptualization, Writing – original draft, Methodology, Investigation, Formal analysis, Conceptualization. **Dongsheng Cai:** Validation, Supervision, Investigation, Data curation. **Humphrey Adun:** Writing – review & editing, Validation, Investigation. **Mustafa Dagbasi:** Writing – review & editing. **Chiagoziem C. Ukwuoma:** Writing – review & editing, Project administration. **Qi Huang:** Writing – review & editing, Validation, Supervision, Investigation. **Nathan Johnson:** Writing – review & editing, Validation, Resources. **Olusola Bamisile:** Writing – review & editing, Validation, Supervision, Methodology, Investigation, Conceptualization.

## Declaration of competing interest

The authors declare that they have no known competing financial interests or personal relationships that could have appeared to influence the work reported in this paper.
